# LOXL2 attenuates osteoarthritis through inactivating Integrin/FAK signaling

**DOI:** 10.1038/s41598-021-96348-x

**Published:** 2021-08-23

**Authors:** Caixia Zhang, Mengjiao Zhu, Huijuan Wang, Juan Wen, Ziwei Huang, Sheng Chen, Hongting Zhao, Huang Li

**Affiliations:** 1 Department of Orthodontics, Nanjing Stomatological Hospital, Medical School of Nanjing University, Nanjing, China; 2 Shanghai Xuhui District Dental Center, 500 Fenglin Road, Shanghai, China; 3 Department of Oral Pathology, Nanjing Stomatological Hospital, Medical School of Nanjing University, Nanjing, China; 4 Medical School of Nanjing University, Nanjing, China

**Keywords:** Drug development, Osteoarthritis

## Abstract

Temporomandibular joint OA (TMJOA) is a common degenerative joint disease, leads to structural damage and ultimately loss of function. Matrix degradation is one of the first pathogenesis during the progression of OA, it was effective to inhibit matrix degradation to block the development of OA. In this study, an in vivo model (compressive mechanical force) and an in vitro model (IL-1β) were used to induce OA-like changes in TMJ cartilage and chondrocytes. We revealed lysyl oxidase like-2 (LOXL2) play a critical role in TMJOA. LOXL2 expression decreased in mechanical stress/IL-β induced TMJOA-like lesions in both in vivo models and in vitro models. Furthermore, recombinant LOXL2 (rhLOXL2) treatment ameliorated the degenerative changes induced by mechanical stress in vivo, including the thinning cartilage, down-expression of collagen II and proteoglycan, and over-expression of TNF-a, while LOXL2 antibody (anti-LOXL2) treatment exacerbated these changes. Mechanistically, the protection of LOXL2 in chondrocytes was induced partly through activation of the Integrin/FAK pathway. The inhibition of the Integrin/FAK pathway could neutralized the effects caused by rhLOXL2. Collectively, our study suggests that the LOXL2 plays a protective role in mechanical stress induced TMJOA-like changes, and the Integrin/FAK pathway may be a key downstream pathway in this process.

## Introduction

Temporomandibular joint osteoarthritis (TMJ OA), the most common TMJ disease, is characterized by progressive degeneration of the articular cartilage^1–3^. Overload compressive mechanical stimulation has been proven to induce OA-like pathological changes in cartilage, including matrix degradation and the death of chondrocytes^4–6^. Although many previous studies have studied the inhibition of chondrocyte death in the treatment of OA^4,7,8^, very few studies focused on the ECM regeneration. However, the onset of TMJ OA may be initially reflected in the ECM surrounding the chondrocytes^9^. Under pathologic conditions, the ECM exhibits a lot of changes associated with increased catabolic activity in the joint^10,11^. Therefore, protection of ECM against pathologic stimulation may be regarded as a new therapeutic target.

LOXL2 belongs to the lysyl oxidase (LOX) protein family (other related members: LOX, LOXL1, LOXL3, and LOXL4^12^. LOXL2 is an extracellular enzyme, which catalyze the oxidative deamination of peptidyl lysine residues, promoting the formation of lysyl-derived cross-linking of collagen and elastin in the ECM. LOXL2 contributs to the tensile strength and structural integrity of many tissues^13^. LOXL2 acts different roles in normal development and diseases, such as promoting the migration and invasion of trophoblasts in preeclampsia^14^ and endothelial-to-mesenchymal transition in angiogenesis^15^ and tumor progression^16^. Furthermore, previous studies found that integrin signaling pathway participated in higher matrix stiffness-induced LOXL2 upregulation in Hepatoma carcinoma cells (HCC)^17^. In cartilage field, some researchers found LOXL2 staining was detected in damaged regions of OA, while not in normal ones^18^. Therefore, the specific role of LOXL2 in cartilage, especially in temporomandibular joint, is need to be identified. We hypothesized that LOXL2 protected cartilage by promoting the cross-linking of collagen to defense the degeneration of ECM in OA.

To investigate this hypothesis, we detected the expression of LOXL2 in an established in vivo animal model of compressive mechanical force loading as reported^4,6^. Then human recombinant LOXL2 (rhLOXL2) and LOXL2 antibody (anti-LOXL2) were used to detect the effect of over/down expression of LOXL2 on the cartilage in TMJ OA. Furthermore, an in vitro model of chondrocytes inflammation induced by IL-1^19,20^ was used to study the mechanism of LOXL2 in TMJ OA like changes. The study provides new insight into the role of LOXL2 in regulating cartilage thinning under mechanical stress and might facilitate the identification of new therapeutic targets for treating TMJ OA diseases.

## Results

### LOXL2 was downregulated in the TMJ OA cartilages induced by compressive mechanical force in vivo

**Figure 1:**
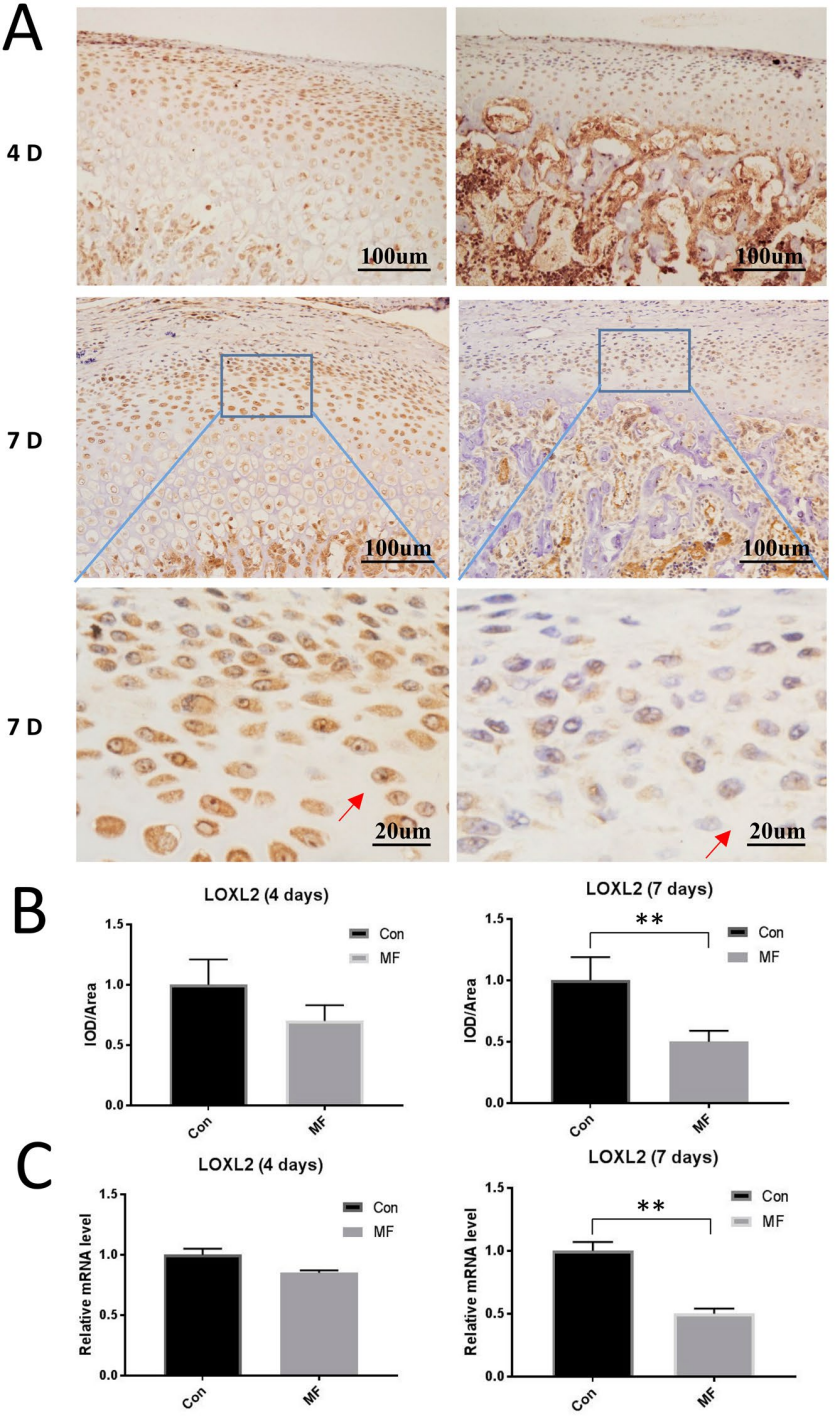
Decreased expression of LOXL2 was found in mechanical stress-mediated cartilage osteoarthritis. (**A**) Immunohistochemical analysis of LOXL2 expression (×100, ×400) and (**B**) quantification of it in chondrocytes from samples treated as indicated at 4 or 7 d after force application (n = 12). Chondrocytes expressing LOXL2 is denoted by arrows. (**C**) RT-qPCR analysis (n = 3) of LOXL2 expression in chondrocytes from samples treated as indicated. Error bars represent S.D. *P < 0.05 versus Con, **P < 0.01 versus Con.

To dissect the underlying function of LOXL2 proteins in the osteoarthritis chondrocytes induced by mechanical stress, we first manipulated a study to assess if LOXL2 expression correlate with the severity of osteoarthritis. We performed immunohistochemistry to detect the localization of LOXL2 proteins in our TMJOA model induced by mechanical force, and we found the expression of LOXL2 decreased after mechanical force. What’s more, LOXL2 expression significantly correlated with the period of mechanical force applied (4 days, p > 0.05; 7 days, p < 0.05). Besides, the results of RT-qPCR also showed that the mechanical force (MF) groups expressed lower levels of LOXL2 compared with the controls (4 days, 18%, p > 0.05; 7 days, 47%, p < 0.05) (Fig. 1). And Positive LOXL2 protein signals was mainly detected within the cytoplasm (Fig. 1A).

### LOXL2 enables the recovery of the degenerative changes induced by mechanical force in vivo

**Figure 2:**
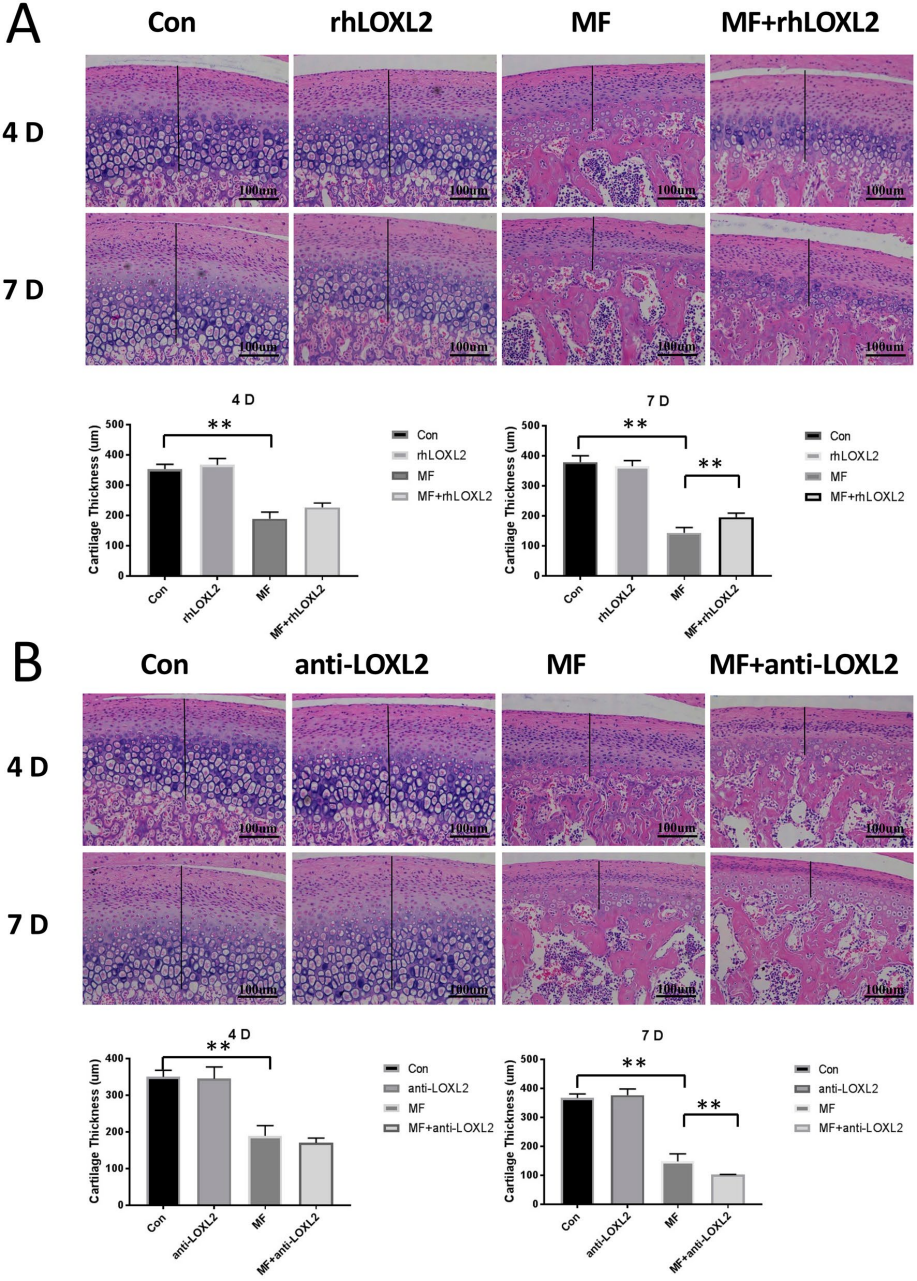
LOXL2 alleviated mechanical force-mediated cartilage thinning. HE-stained cartilage sections were treated as indicated (n = 12). (**A**) Cartilage thinning in MF groups was recovered by rhLOXL2, (**B**) while worsened by anti-LOXL2. The scale bar indicates 100 μm. Cartilage thickness is indicated by a black bar. Quantification of cartilage thickness was listed below the HE-stained images (n = 12). *P < 0.05, **P < 0.01.

To investigate the specific importance of LOXL2 in the progress of cartilage degenerative changes induced by mechanical stress, we measured the changes of the thickness of TMJ cartilage under the treatment of exogenous (rhLOXL2) or inhibited LOXL2 (LOXL2 antibody, anti-LOXL2) with mechanical force in vivo. As our previous studies, the application of compressive mechanical force resulted in cartilage thinning at 4 days (190.46 ± 11.32 μm, *p* < 0.01) and 7 days (142.83 ± 11.74 μm, *p* < 0.01) compared with the controls (350.21 ± 10.31 μm, 4 days; 367.32 ± 9.89 μm, 7 days. The thinning cartilage recovered by rhLOXL2 in the 4 days (220.33 ± 8.73 μm, + 16%, *p* > 0.05) and 7 days (195.31 ± 6.34 μm, + 37%, *p* < 0.01) group, while escalated after anti-LOXL2 treatment in the 4 days (170.63 ± 5.39 μm, − 10%, *p* > 0.05) and 7 days (103.87 ± 5.52 μm, − 27%, *p* < 0.01) group. And no significant changes in cartilage thickness were observed when rhLOXL2 or anti-LOXL2 was treated alone (Fig. 2).

### LOXL2 Increases Collagen Accumulation and suppresses inflammation in TMJOA cartilage Induced by mechanical stress

**Figure 3:**
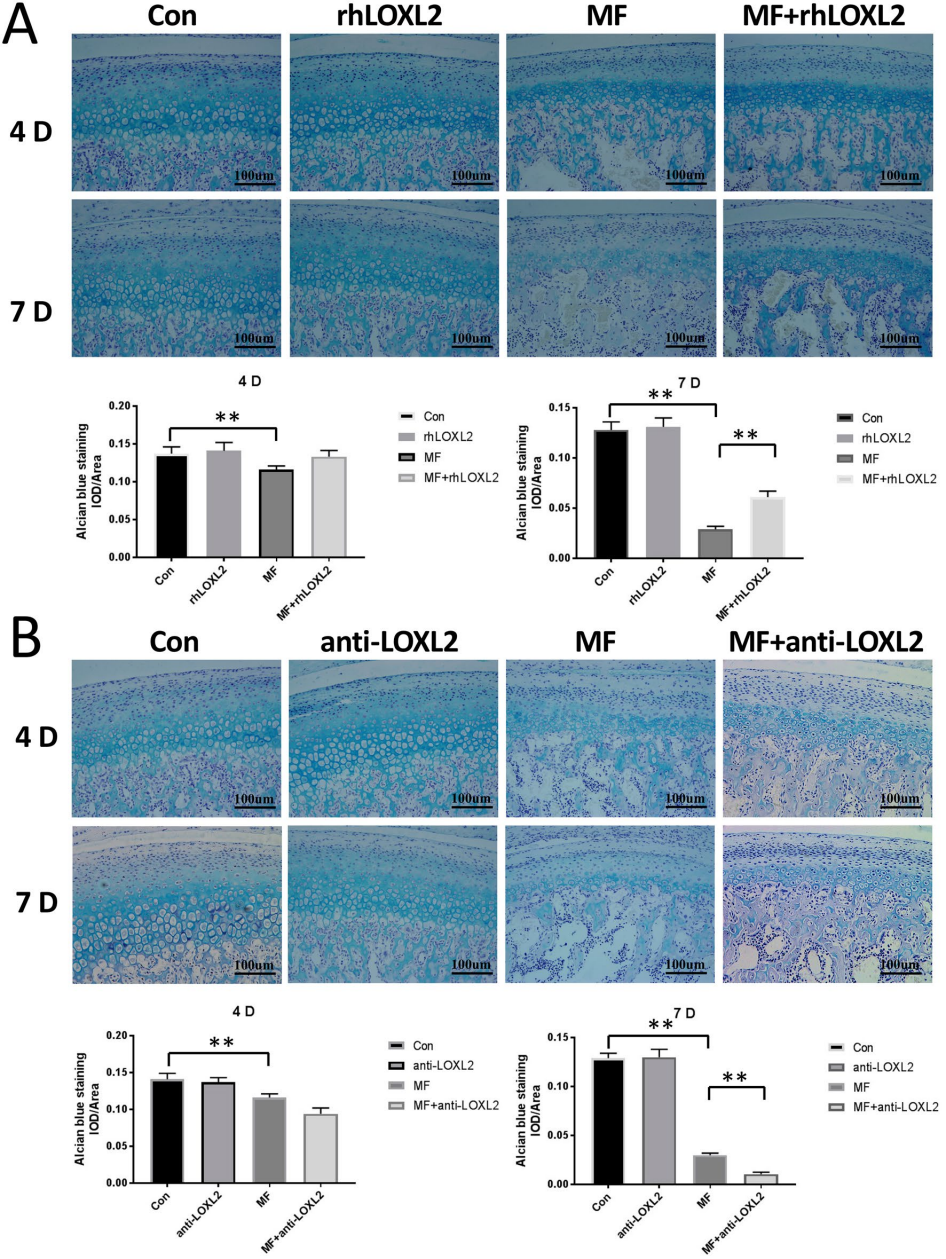
LOXL2 restored ECM degradation in mandibular condyle cartilage at 4 and 7 d after force application. Alcian blue-stained cartilage sections were treated as indicated (n = 12). (**A**) Degradation of proteoglycan in F groups was recovered by rhLOXL2, (**B**) while worsened by anti-LOXL2. Quantification was listed below (n = 12). *P < 0.05, **P < 0.01.

Considering the role of LOXL2 proteins in ECM remodeling and collagen cross-linking, we speculated that the protecting role of LOXL2 under mechanical stress of TMJ was due to modulating collagen accumulation. To test this hypothesis, we detected proteoglycans by alcian blue staining and collagen II expression by immunohistochemical staining in the presence or absence of added rhLOXL2 or anti-LOXL2. The results of Alcian blue staining showed that there were significant decreases of proteoglycan’s expression after 4-days (− 33%, p < 0.05) or 7-days (− 77%, p < 0.05) mechanical stress. And the decreasing expression recovered by rhLOXL2 in the 4-days (+ 15%, *p* > 0.05) or 7-days (+ 108%, *p* < 0.05) groups compared with the MF groups, while get worse after anti-LOXL2 treatment in the 4 days (− 19%, *p* > 0.05) and 7-days (− 65%, *p* < 0.05) group compared with the 4/7-days MF groups. And no significant changes were observed when rhLOXL2 or anti-LOXL2 treatment was used alone (Fig. 3).

**Figure 4:**
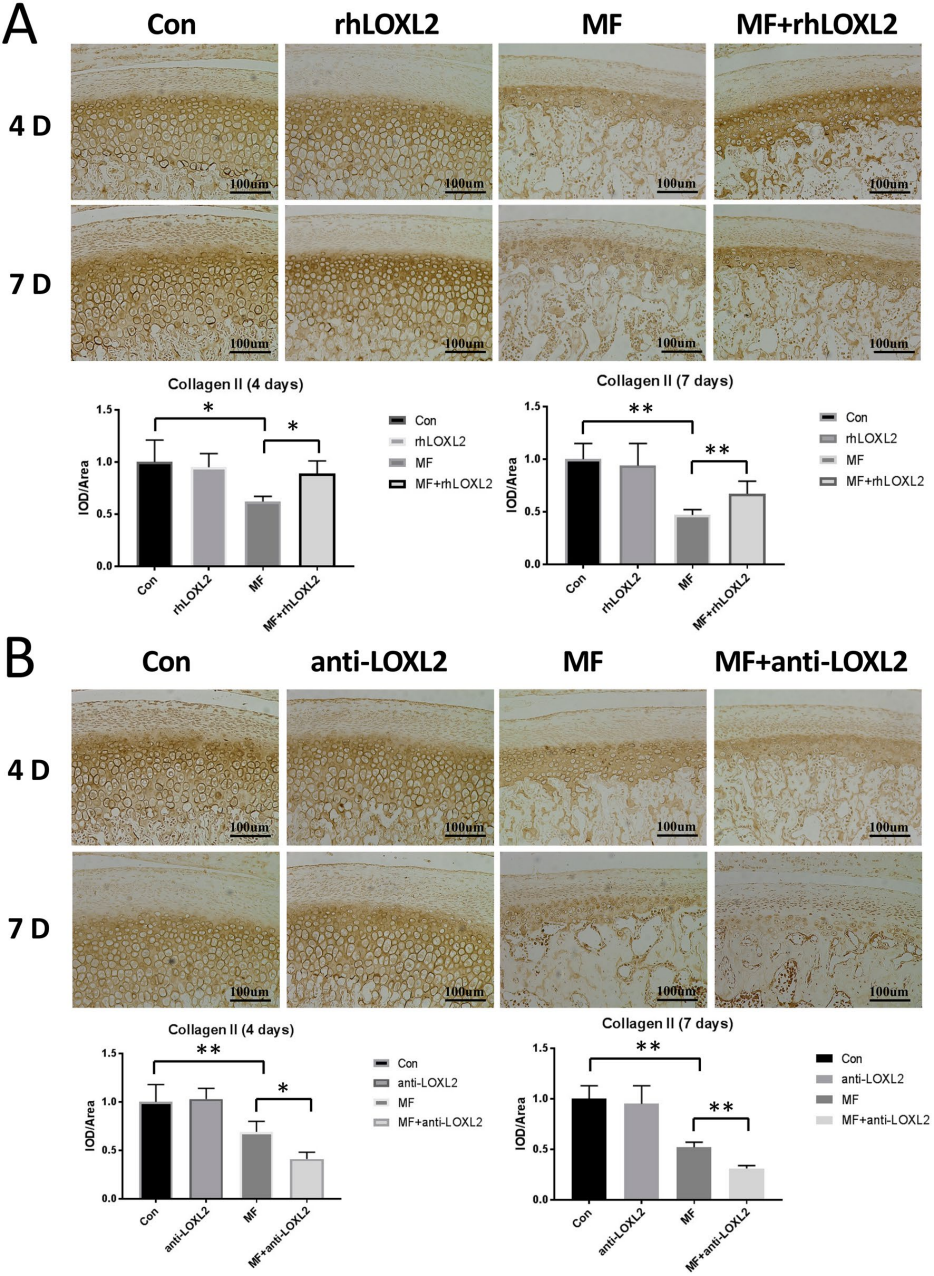
LOXL2 restored collagen II degradation in mandibular condyle cartilage at 4 and 7 d after force application. Immunohistochemical analysis of Collagen II expression and quantification of it in chondrocytes from samples treated as indicated (n = 12). (**A**) Degradation of collagen II in MF groups (4-days, p < 0.05; 7-days, p < 0.01; compared with the controls) was recovered by rhLOXL2(4-days, p < 0.05; 7-days, p < 0.01; compared with the MF groups), (**B**) while worsened by anti-LOXL2(4-days, p < 0.05; 7-days, p < 0.01; compared with the MF groups).

Consistent with the changes of proteoglycan’s expression, the immunohistochemical staining indicated that decreased expression of collagen II induce by mechanical force, which was correlated with the period of mechanical force applied (4-days, p < 0.05; 7-days, p < 0.01; compared with the controls) was increased by rhLOXL2 (4-days, p < 0.05; 7-days, p < 0.01; compared with the MF groups), while decreased by anti-LOXL2 (4-days, p < 0.05; 7-days, p < 0.01; compared with the MF groups) (Fig. 4). In parallel, RT-qPCR results also showed the decreasing expression of LOXL2 induced by mechanical force was ameliorated by rhLOXL2 (p < 0.05) (Appendix Fig. 2a). Taken together, these results indicated that the expression of ECM component was protected by LOXL2 in cartilage under mechanical stress.

**Figure 5:**
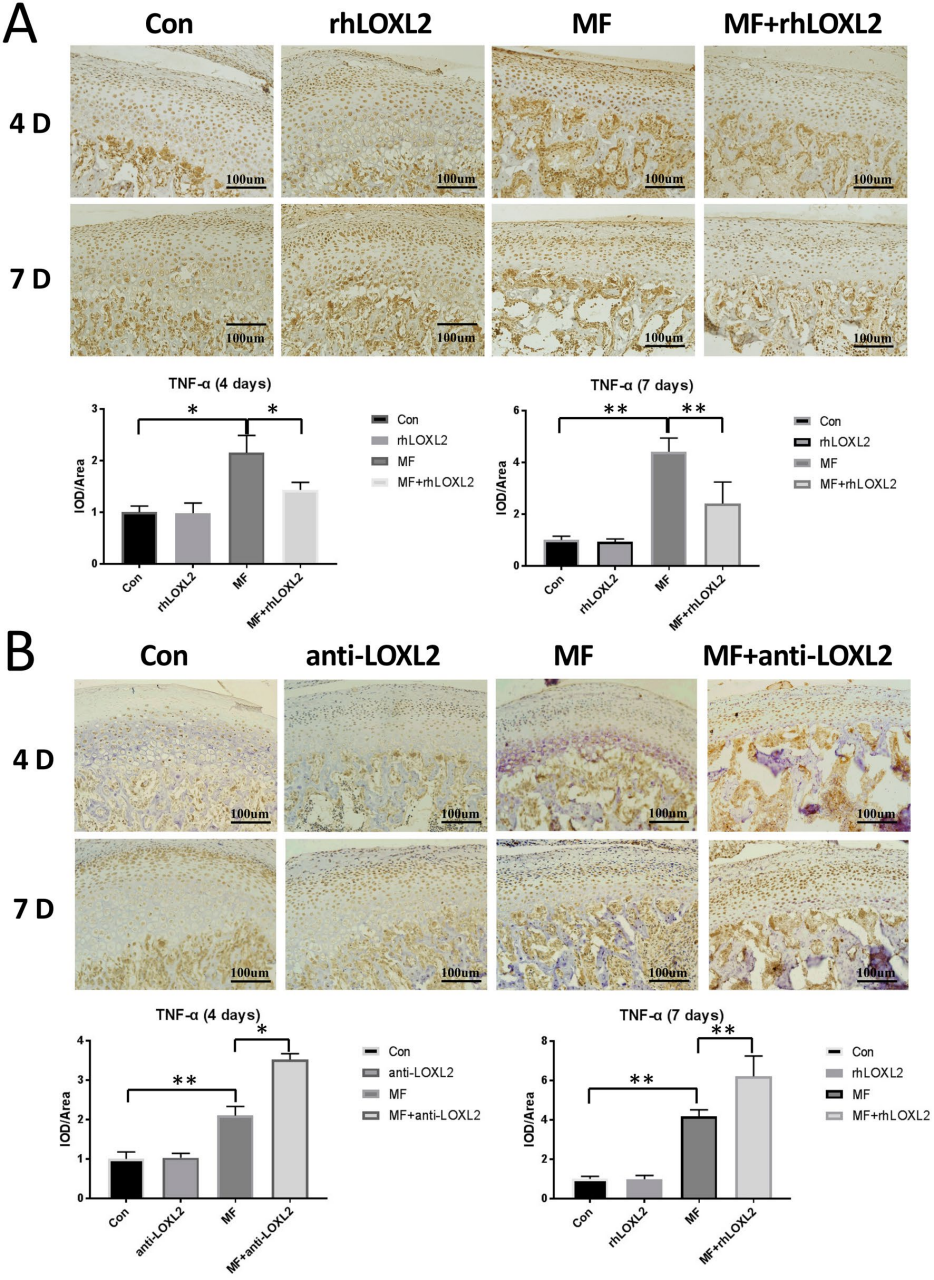
LOXL2 restored TNF-a over-expression in mandibular condyle cartilage at 4 and 7 d after force application. Immunohistochemical analysis of TNF-a expression and quantification of it in chondrocytes from samples treated as indicated at 4 or 7 d after force application (n = 12). (**A**) Degradation of TNF-a in MF groups (4-days, p < 0.05; 7-days, p < 0.01; compared with the controls) was recovered by rhLOXL2 (4-days, p < 0.05; 7-days, p < 0.01; compared with the MF groups), (**B**) while worsened by anti-LOXL2 (4-days, p < 0.05; 7-days, p < 0.01; compared with the MF groups).

Following the observation that TMJ cartilage thinning and ECM degeneration induced by mechanical stress could be attenuated by LOXL2, we sought to determine whether LOXL2 could inhibit the expression of inflammation factors, which was another important manifestation of TMJ OA. As our previous studies, TNF-a expression was significantly upregulated in the protein (4-days, p < 0.05; 7-days, p < 0.01) and mRNA (4-days, p < 0.05; 7-days, p < 0.05) level after 4- or 7-days mechanical stress compared with the controls. And rhLOXL2 could attenuate its expression in the protein (4-days, p < 0.05; 7-days, p < 0.01) and mRNA (4-days, − 36%, p < 0.05; 7-days, − 47%, p < 0.05) level compared with the MF groups, while anti-LOXL2 could worsen it (4-days, p < 0.05; 7-days, p < 0.01; compared with the MF groups). In hence, LOXL2 also acted an anti-inflammation role in cartilage under mechanical stress (Fig. 5 and Appendix Fig. 2b).

### LOXL2 activated Integrin-FAK signaling in mandibular chondrocytes

**Figure 6:**
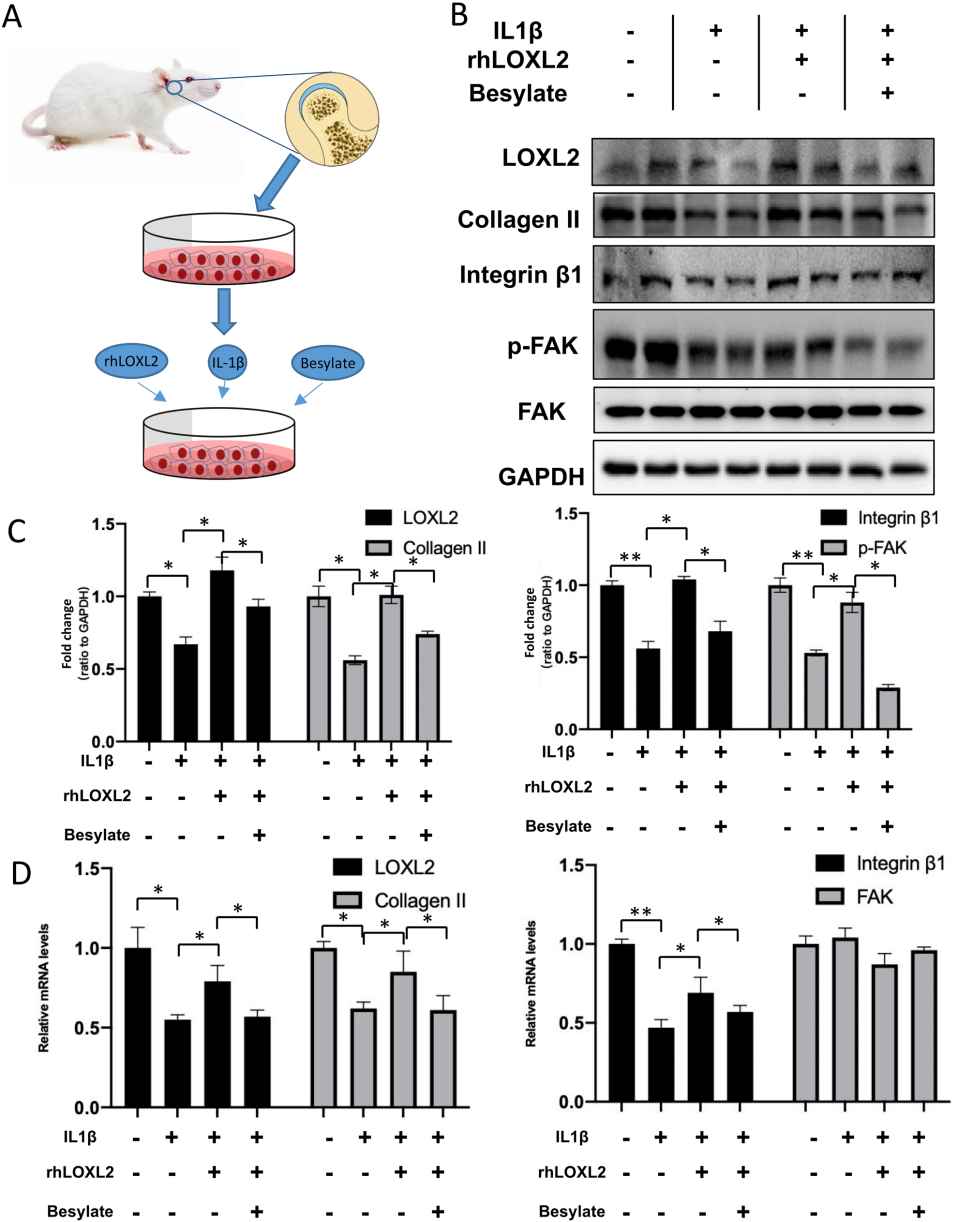
LOXL2 restored IL-1β-mediated Collagen II degradation through Integrin/FAK pathway. (**A**) Flow chart of the isolation of TMJ chondrocytes and schematic diagram of agent-treated chondrocytes. (**B**) Western blot analysis and (**C**) quantitative results of LOXL2, Collagen II, Integrin β1, p-FAK and FAK expression in chondrocytes from samples treated as indicated. (**D**) RT-qPCR analysis of LOXL2, Collagen II, Integrin β1 and FAK expression in chondrocytes from samples treated as indicated. Error bars represent S.D. *P < 0.05, **P < 0.01.

Integrins has been reported to be a transmembrane protein monitoring and communicating with ECMs in chondrocytes, relaying chemical and mechanical stimuli between the outside and inside of cells. These findings prompted us to explore whether LOXL2 modulates collagen expression in chondrocytes through the integrins pathway. Primary mandibular chondrocytes were treated with *IL-1*β alone or in combination with rhLOXL2, and molecular signaling and gene expression were assessed (Fig. 6A). 10 ng/L *IL-1*β reduced mRNA levels of LOXL2 by − 54% (p < 0.05), and 100 ng/ml rhLOXL2 could ameliorated the decrease of Integrin β1 and p-FAK induced by *Il-1β* by + 18% (p < 0.05) and + 22% (p < 0.05) (Appendix Fig. 3).

To determine whether integrin and its downstream FAK signaling mediate chondrocytes in response to exogenous LOXL2, we used specific FAK inhibitors PF-00562271 Besylate. And the results showed that the recovery effect of rhLOXL2 compared with *IL-1*β treated alone was blunted by Besylate. The increased expression of LOXL2 (+ 75%, p < 0.01) and Collagen II (+ 80%, p < 0.01) by *IL-1*β + rhLOXL2 treatment compared with *IL-1*β treated alone was reduced by Besylate by − 20% (p < 0.05) and − 24% (p < 0.05). What’s more, Besylate could also suppress the recovery expression of integrin β1 (+ 87%, p < 0.01) and p-FAK (+ 66%, p < 0.01) induced by rhLOXL2 by − 35% (p < 0.05) and − 69% (p < 0.05) (Fig. 6B,C). and the results of RT-qPCR were in accordance with it (Fig. 6D). Thus, LOXL2 may protect against the degeneration changes of TMJ cartilage induced by mechanical stress through Integrin-FAK signal.

## Discussion

Osteoarthritis is a progressive joint disease which affects a significant portion of the population but has a few therapeutic options. Degeneration of exocellular matrix is thought to be a significant pathological change in OA. In this study, we uncovered that LOXL2 acted as a protective factor in TMJ OA induced by compressive mechanical stress. We found decreased expression levels of LOXL2 co-related with the time of mechanical force in cartilage. The molecular mechanism was demonstrated by LOXL2 modulation of ECM expression, cartilage thickness and inflammation, and Integrin-FAK signaling pathway participated in this progress.

The ECM is maintained in a balance of anabolism and catabolism of matrix synthesis and degradation in healthy articular cartilage. However, under pathologic conditions, for example excessive mechanical stress, the biological imbalance lead to a pathological situation with altered chondrocytes behavior releasing inflammatory mediators and ECM destruction enzymes^21^. Previous studies mainly focused on the inhibition of the death of chondrocytes^7,8^, there are few studies about the regeneration of ECM.

LOXL2 function is critical for normal chondrogenic differentiation^22^. It was reported that LOXL2 expression is increased during endochondral ossification in a fracture healing model in mice^18^. In our model, we found that LOXL2 expression is decreased in acute TMJ OA models mediated by mechanical stress in vivo and IL-1β induced inflammation changes in chondrocytes, exogenous LOXL2 could rescue the thinning cartilage, increase the expression of aggrecan and collagen II. LOXL2 could be a potential protective factor in OA cartilage that could lead to cartilage regeneration. Furthermore, we noticed the recovery of proteoglycan and collagen II expression was more obviously than the cartilage thickness, reminding the function of promoting matrix crosslinking of LOXL2^13^. The protection to ECM was earlier than the recovery of the thinning of cartilage thickness, which supported the TMJ OA was initially occurred in the ECM in turn. Besides the regeneration of ECM, LOXL2 attenuated the over-expression of TNF-α in rats TMJ cartilage stimulated by mechanical stress, consistent with other studies^18^. The anti-inflammatory role of LOXL2 might be the secondary pro-regenerative function after matrix crosslinking, and LOXL2 is expected to serve as a therapeutical target in TMJOA.

Integrin is a transmembrane protein relaying chemical and mechanical stimuli between the outside ECMs and inside of cells. The integrin-mediated signaling cascade is one of the most critical signaling pathways for cartilage development and function^23^. LOXL2 is a potent regulator of integrin α5 and integrin β1 protein levels in clinical clear cell renal cell carcinoma specimens, and the expression levels of LOXL2 and integrin α5 correlated with the pathologic tumor grades^24^. And in HCC cells, LOXL2 upregulation induced by higher matrix stiffness was associated with Integrin signaling pathway^17^. In the present study, we found that the Integrin-FAK pathway was activated by rhLOXL2, and the recovery effect of rhLOXL2 was partly blunted by FAK inhibitors. Also, inhibition of FAK could partially neutralize the protection of rhLOXL2. Therefore, the Integrin-FAK signal pathway might be a key downstream pathway of LOXL2 in TMJ OA.

## Conclusion

In our present study, we introduced a protective factor-LOXL2, which could promote the ECM regeneration of TMJ cartilage under mechanical stress. And Integrin-FAK signaling pathway participated in this progress. Our data suggest a potential novel pharmaceutical target for the treatment of TMJ OA.

## Materials and methods

### Animals

A total of 84 (the power calculated by PASS 11.0 software was more than 80%) 7-week-old male Sprague–Dawley (SD) rats were requisitioned in this study. Experimental protocols complied with the ARRIVE (Animal Research: Reporting in Vivo Experiments) guidelines for preclinical animal studies, and were approved by the Animal Care and Use Committee of Nanjing University. All experiments were performed in accordance with relevant guidelines and regulations. And all animals were obtained from the Model Animal Research Center of Nanjing University, and housed in the specific-pathogen-free laboratory animal room at Nanjing University.

After acclimatizing to the laboratory conditions with food and water available adlibitum for 1 week, all rats were randomly divided into two groups: non-mechanical force (n = 42) and mechanical force (MF) groups (n = 42; 4-day and 7-day treatments), with gender- and age-matched controls according to our previous study (Appendix Fig. 1). The rats in the MF group were exerted with 80 g of compressive mechanical force created by a rubber band tied between a jig and anchorage hooks for 4 or 7 days as described previously. After applied for 24 or 96 h in the 4- or 7-day groups, human recombinant LOXL2 (rhLOXL2, R&D Systems, 2639-AO-010, USA, 0.44 μg, a 0.2 μm filtered solution in MES and NaCl) or LOXL2 antibody (anti-LOXL2, Santa Cruz, sc-48724, USA, 2 μg, a 100 µg/ml solution in PBS) were injected locally into the TMJ on one side, and a vehicle (dimethyl sulfoxide; Solarbio) was injected on the other side, as described previously. After 4 or 7 days, the rats were sacrificed by cervical dislocation under anesthesia, and 2 rats displayed signs of disability (Appendix Fig. 1).

### Histological observation and histomorphometric measurements

Hematoxylin and eosin (HE)-staining was conducted as described previously. The condyles were harvested with the surrounding tissue. After fixed in 4% formalin for 24 h and decalcified in 15% ethylenediaminetetraacetic acid (EDTA) solution for 8 weeks, the specimens were embedded in paraffin after a thorough rinsing. Then, 5-μm-thick sagittal sections were cut from each embedded TMJ block parallel to the lateral surface of the condylar neck of the mandible ramus. The paraffin sections were deparaffinized in xylene and rehydrated in a graded alcohol series. Then, the samples were stained by hematoxylin, followed by counterstaining of eosin. Images were captured with an Olympus XI 70 microscope equipped with an Olympus Magna Fire digital camera. Thickness measurement were determined using a computer-assisted image analysis system (Image-Pro Plus, version 6.0; Media Cybernetics) at the same staining threshold. The thresholds for color balance, brightness, and texture were adjusted to best optimize all images to be compared. Condylar thickness was measured on three HE-stained sections per joint, and the average values were used for the statistical analyses (n = 12).

### Alcian Blue staining

For Alcian Blue staining, samples were stained by Alcian Blue for 20 min after deparaffinization and rehydration as above. After soaked for 2 min in 3% ethylic acid solution, the specimens were then counterstained under nuclear fast red for 5 min. Images were acquired as above for the HE staining sections. Three areas were chosen in the mandibular condylar cartilage. IOD/Area measurement were determined using a computer-assisted image analysis system (Image-Pro Plus, version 6.0; Media Cybernetics) at the same staining threshold as above (n = 12).

### Immunohistochemical staining

Immunohistochemical staining was conducted as described previously^4^. After treated with 3% hydrogen peroxide to eliminate endogenous peroxidase activity and with Antigen Retrieval Solution (Wuhan Boster Biological Technology Ltd. China) to digest the antigenic sites, the sections were incubated overnight with anti-LOXL2 (1:400, Santa Cruz, sc-48724, USA), anti-TNF-a (1:1000, ab6671, Abcam, USA), or anti-Collagen II (1:100, KG22256, KeyGEN Biotech) at 4 °C. Next, the specimens were incubated with biotin-labeled IgG (BeiJing ZhongShan Golden Bridge Biotechnology Co., China) for 30 min at 37 °C and an avidin-peroxidase complex for 30 min at 37 °C. Then, the specimens were incubated with a peroxidase/diaminobenzidine (DAB) yellow kit (Wuhan Boster Biological Technology Ltd., China) to stain antibody, and counterstained with hematoxylin, dehydrated in an ethanol series, cleared in xylene and cover slipped. Images were acquired as above for the HE staining sections. A color difference was used to delineate positive and negative areas, which were measured with Image-Pro Plus software.

### Mandibular chondrocytes culture

Primary mandibular chondrocytes were isolated from 3-week-old male SD rats TMJ as described previously (Fig. 6A). The cultured primary mandibular chondrocytes were divided into 6 groups: control (CON), IL-1β (cells treated with 10 ng/mL IL-1β (Sigma, US)), rhLOXL2 (cells treated with 100 ng/mL rhLOXL2 (R&D Systems, 2639-AO-010, USA)), Besylate (cells treated with 1.5 nM PF-00562271 Besylate (Selleck, S2672, USA)), IL-1β + rhLOXL2, and IL-1β + rhLOXL2 + Besylate. Cells were pretreated with rhLOXL2 and Besylate for 12 h before treatment with IL-1β for another 24 h. The cells were viewed on a microscope (Nikon ECLIPSE TS100, Japan) equipped with a camera and image-capture software.

### Isolation of total RNA and RT-qPCR

Total RNA was isolated using TRIzol (Invitrogen Life Technologies, CA, USA) as described previously. Three condyles from three different rats were used as a single cartilage sample for RNA extraction in the in vivo model, and different groups of cells from a 6-well plate were collected in the in vitro model. The primers used are listed in Table 1.

**Table 1:** Primers used in real-time RT-PCR.

Gene	Primer sequences (5′–3′)
GAPDH	Fwd: 5′-AAAGGCATCTTGGGCTACACCG-3′
	Rev: 5′-ATGAGGTCCACCACCCTGTTG-3′
LOLX2	Fwd: 5′-CGATTGCCACCTCCTTGCTA-3′
	Rev: 5′-CCAGAGCCCTGCCCCTAA-3′
Collagen II	Fwd: 5′-AGAGCGGAGACTACTGGATTG-3′
	Rev: 5′-TCTGGACGTTAGCGGTGTT-3′
TNF-a	Fwd: 5′-CCACGCTCTTCTGTCTACTG-3′
	Rev: 5′-GCTACGGGCTTGTCACTCGA-3′
Integrin β1	Fwd: 5′-AATTCAAGAGGGCTGAAGACTAC-3′
	Rev: 5′-TGTCAGTAAGACTAAGCACG-3′
FAK	Fwd: 5′-CCAAGTTCGAGTACTAAGACTCACC-3′
	Rev: 5′-AAATCCATAGCAGGCCACGTGC-3′

### Western blotting

Western blotting was conducted as described previously^4^. The incubated overnight antibodies include rabbit anti-LOXL2 (1:1000, Santa Cruz, sc-48724, USA), rabbit anti-Integrin β1 (1:1000, Abcam, ab179471, USA), rabbit anti-p-FAK (1:1000, Abcam, ab81298, USA), and rabbit anti-collagen II (1:1000, Abcam, ab185430, USA). The membranes were visualized using an ECL Plus Kit (Amersham Biosciences, Amersham, Bucks, UK) and quantified by densitometry.

### Statistical analysis

Statistical analysis was conducted using 1-way ANOVA-tests. All the data were analyzed with SPSS 16.0 software, and *p* < 0.05 was defined as statistically significant. All measurements were repeated three times. The data was represented as means + standard deviations.

## Supplementary Information

Supplementary Information.
