# Supplementary material for: LOXL2 attenuates osteoarthritis through inactivating Integrin/FAK signaling
Source: Sci Rep. 2021 Aug 23;11:17020. doi: 10.1038/s41598-021-96348-x (PMC8382747; doi:10.1038/s41598-021-96348-x)
Supplement: Supplementary file 1 — Supplementary Information. [file 41598_2021_96348_MOESM1_ESM.pdf]

LOXL2 attenuates osteoarthritis through inactivating Integrin/FAK signaling

Caixia Zhang[1¶], Mengjiao Zhu[2¶], Huijuan Wang[1], Juan Wen[1], Ziwei Huang[1], Sheng Chen[3], Hongting Zhao[4], Huang Li[1*]

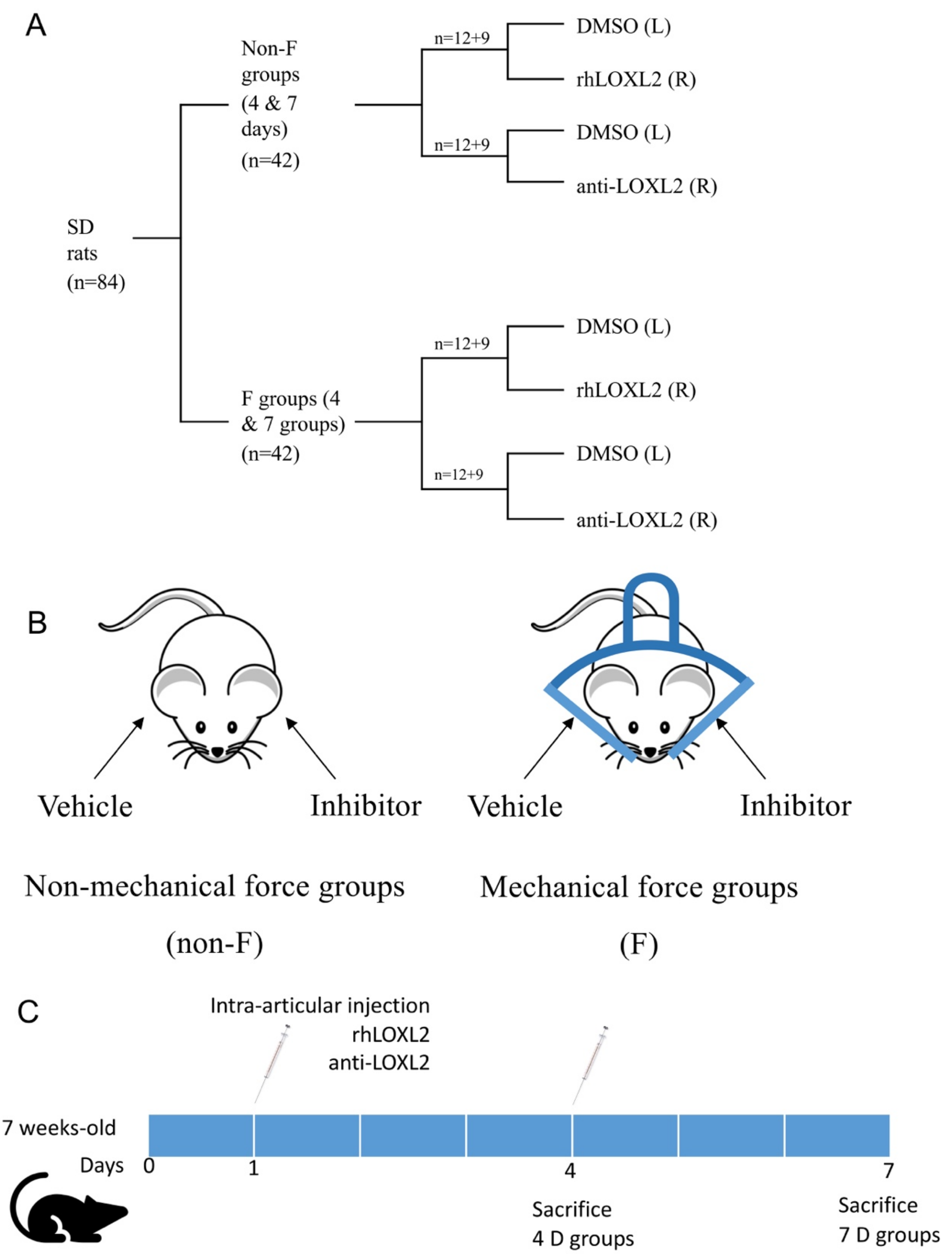

Appendix Figure 1. (A) Detailed description of the 4-day and 7-day groups. (B) & (C)Diagram of the experimental design referred to in A.

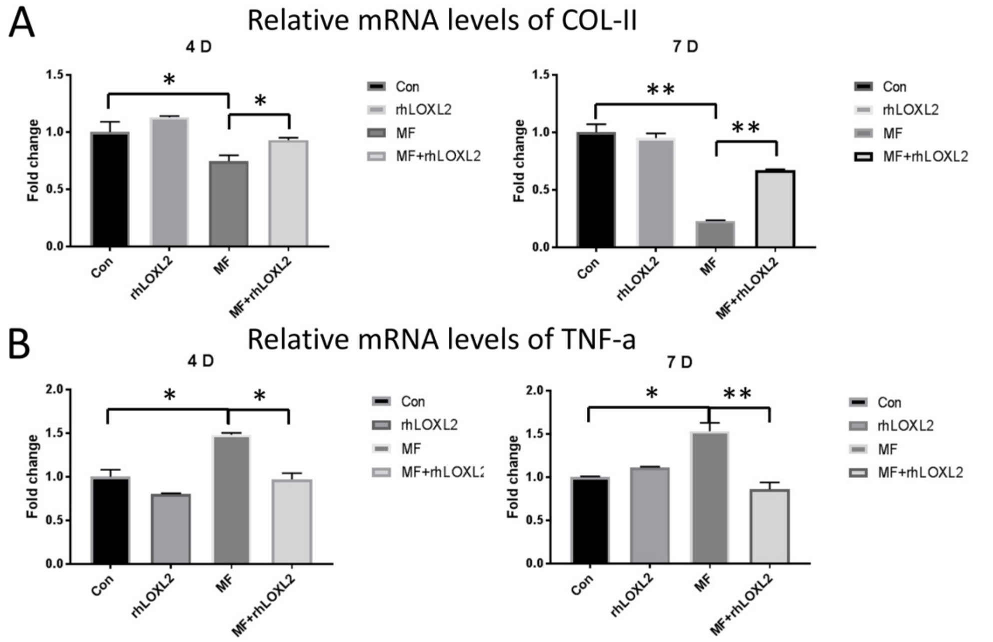

Appendix Figure 2. RT-qPCR analysis of (A) Collagen II and (B) TNF-a expression in chondrocytes from samples treated as indicated at 4 or 7 d after force application (n=3). *P < 0.05, **P < 0.01.

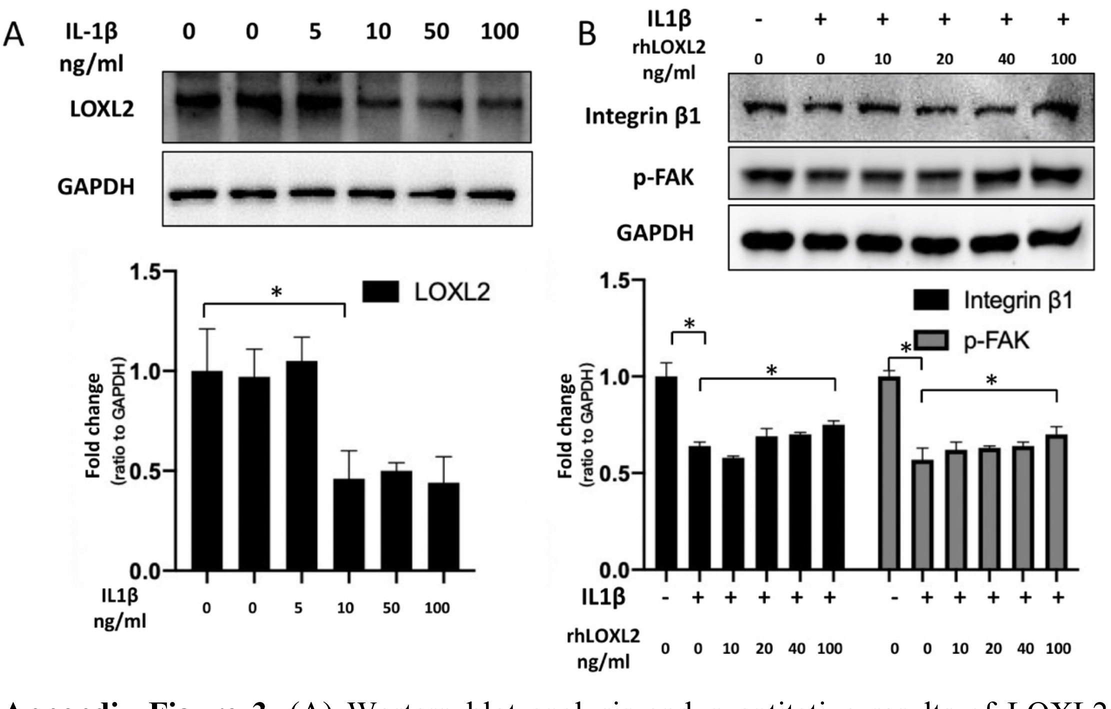

Appendix Figure 3. (A) Western blot analysis and quantitative results of LOXL2 expression in chondrocytes from samples treated as indicated. (B) Western blot analysis of Integrin β1 and p-FAK expression in chondrocytes from samples treated as indicated. Error bars represent S.D. *P < 0.05.

Original images for western blot.

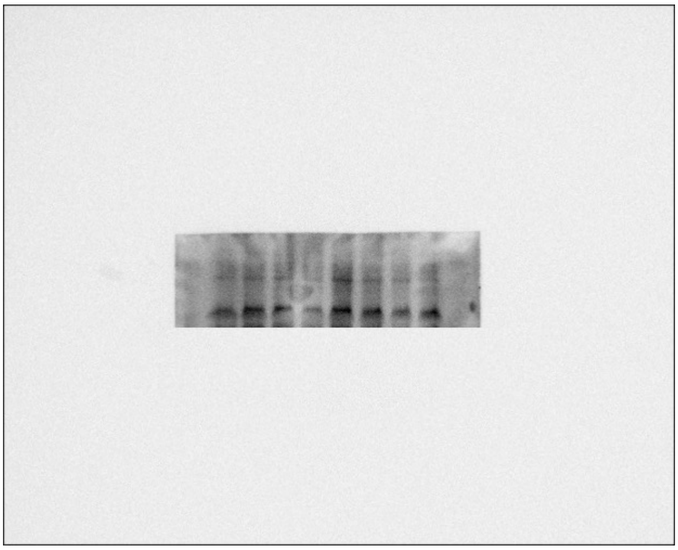

Figure 6 LOXL2

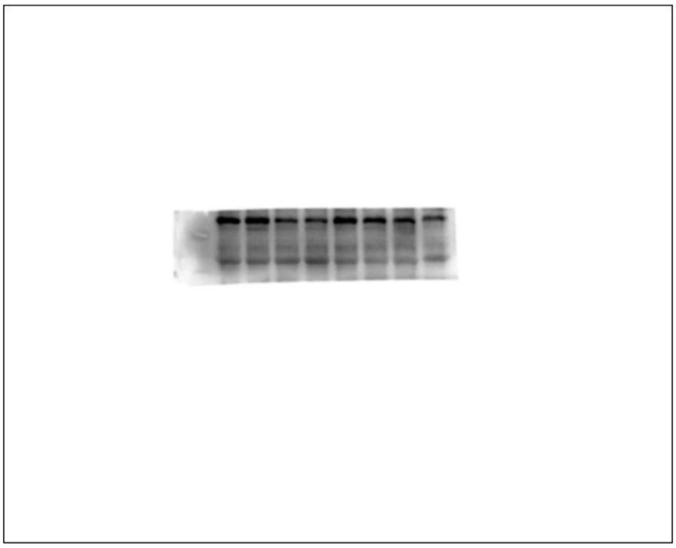

Figure 6 Collagen II

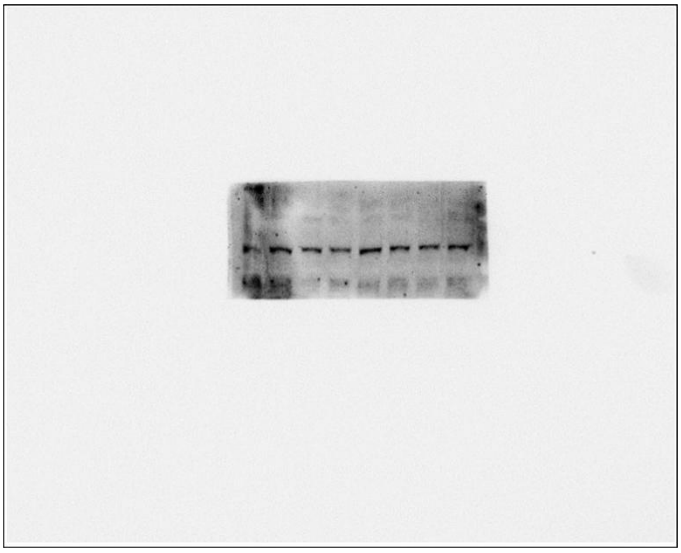

Figure 6 Integrin β1

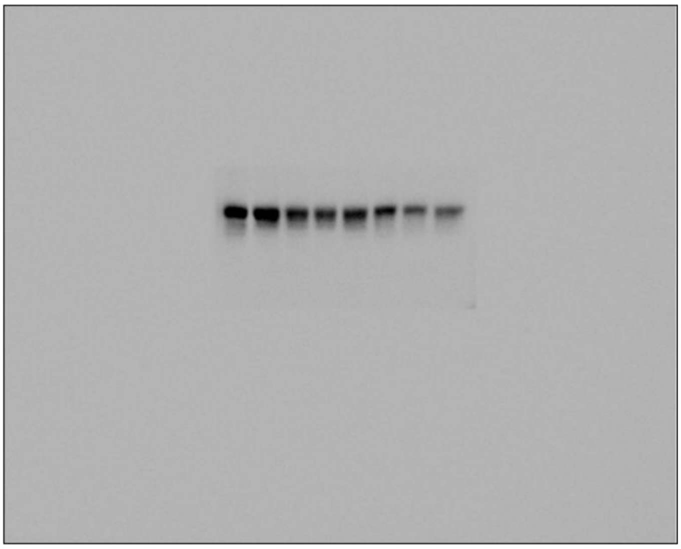

Figure 6 p-FAK

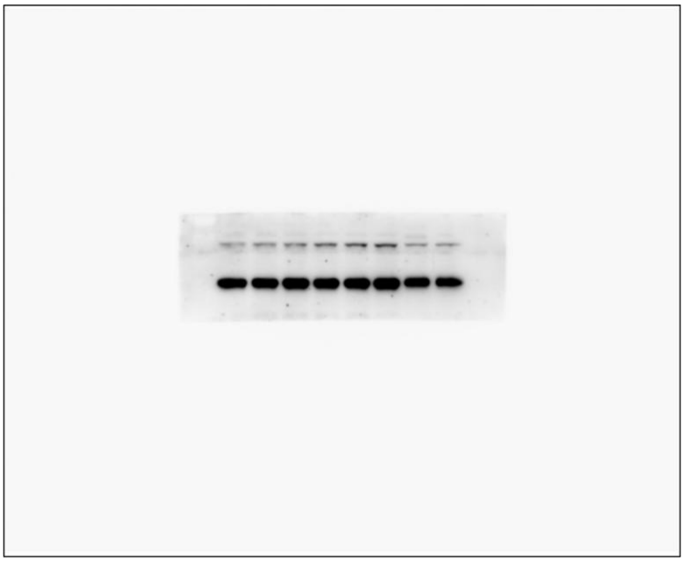

Figure 6 FAK

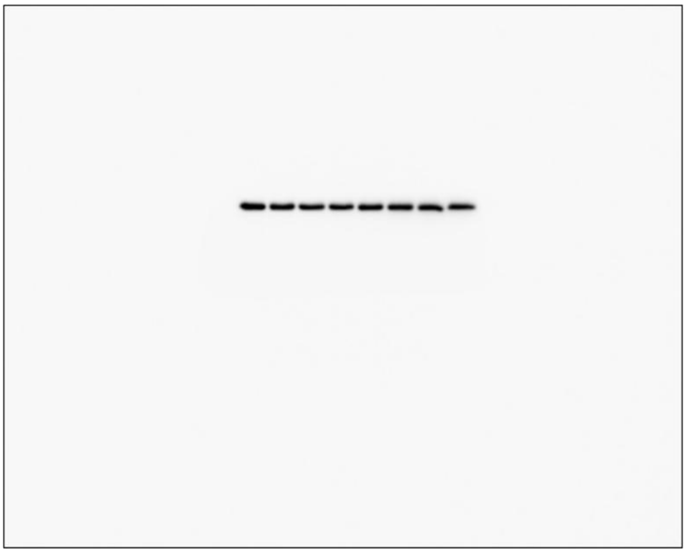

Figure 6 GAPDH-1

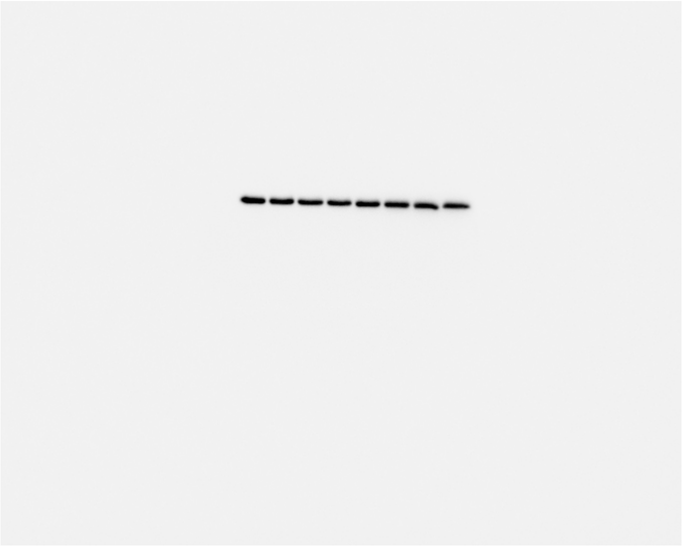
GAPDH-2

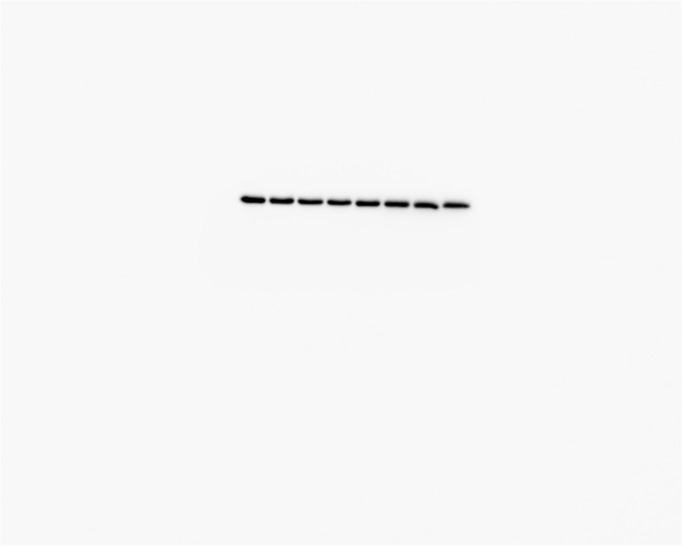
GAPDH-3

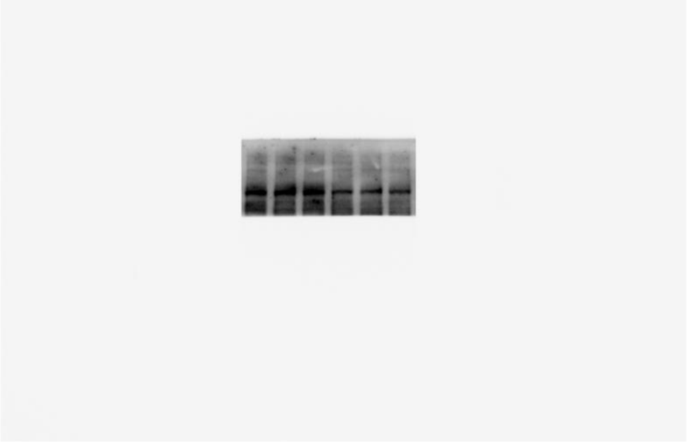
Appendix Figure 3 A LOXL2

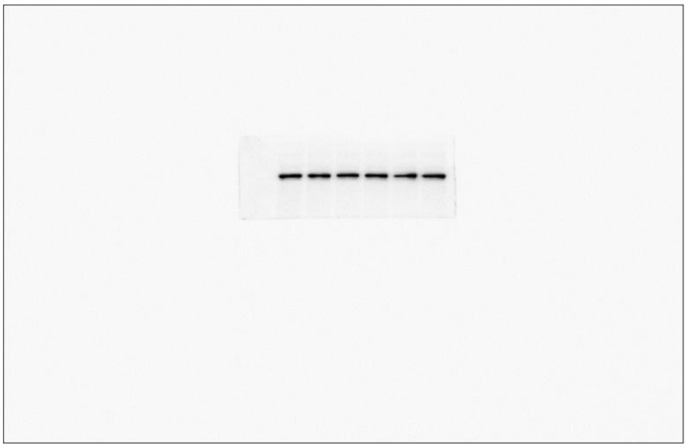

Appendix Figure 3 A GAPDH

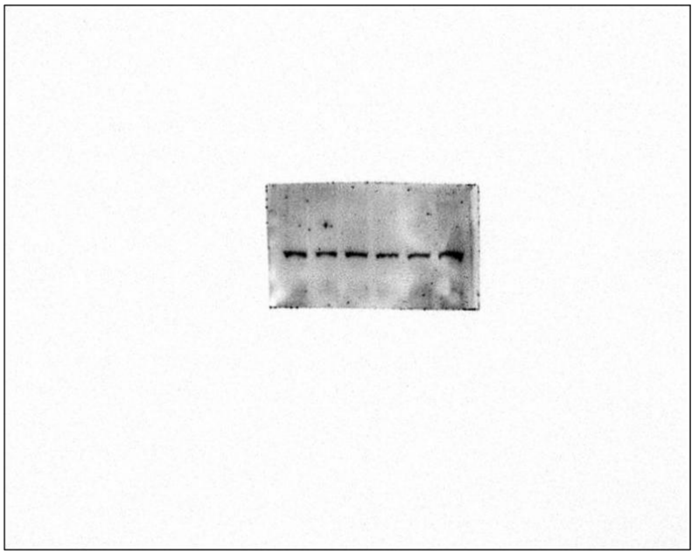

Appendix Figure 3 B Integrin $\beta 1$

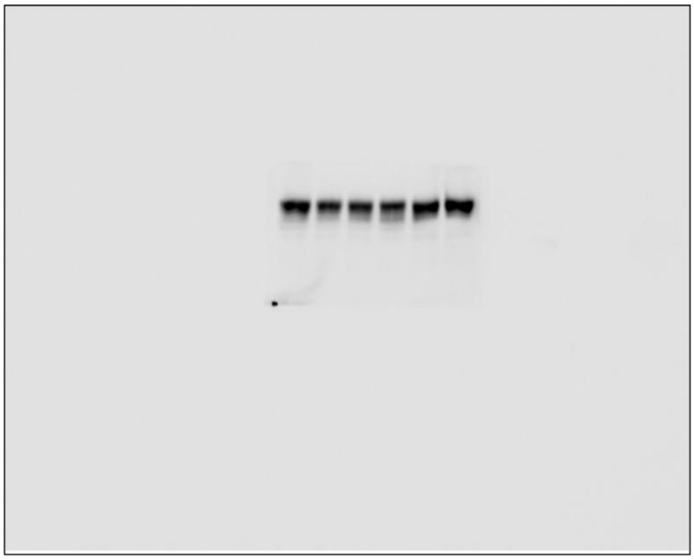

Appendix Figure 3 B p-FAK

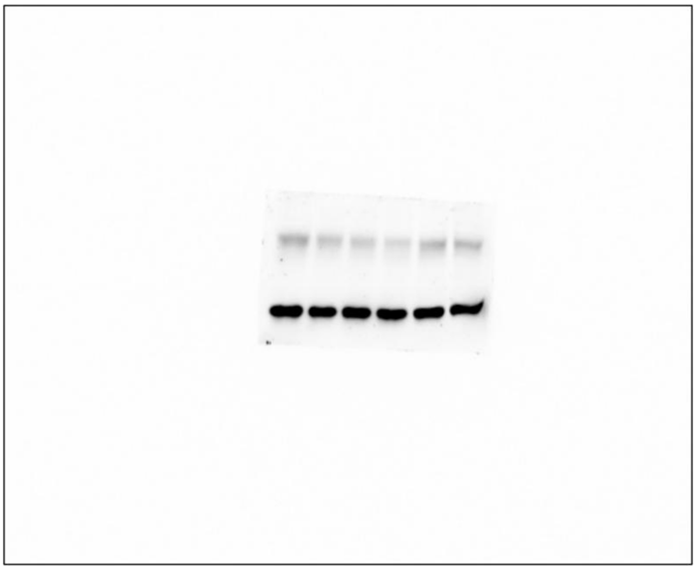

Appendix Figure 3 GAPDH
